# Anti-Inflammatory Activities of a Chinese Herbal Formula IBS-20 *In Vitro* and *In Vivo*

**DOI:** 10.1155/2012/491496

**Published:** 2012-02-22

**Authors:** Zhonghan Yang, Viktoriya Grinchuk, Siu Po Ip, Chun-Tao Che, Harry H. S. Fong, Lixing Lao, Justin C. Wu, Joseph J. Sung, Brian Berman, Terez Shea-Donohue, Aiping Zhao

**Affiliations:** ^1^Department of Medicine and Mucosal Biology Research Center, University of Maryland School of Medicine, Baltimore, MD 21201, USA; ^2^Department of Biochemistry, Zhongshan Medical School, Sun Yat-sen University, 74 Zhongshan 2nd Road, Guangzhou 510080, China; ^3^School of Chinese Medicine, The Chinese University of Hong Kong, Hong Kong; ^4^Department of Medicinal Chemistry and Pharmacognosy, University of Illinois at Chicago, Chicago, IL 60612, USA; ^5^Center for Integrative Medicine, University of Maryland School of Medicine, Baltimore, MD 21201, USA; ^6^Institute of Digestive Disease, The Chinese University of Hong Kong, Hong Kong

## Abstract

Irritable bowel syndrome (IBS) is a functional bowel disorder and the etiology is not well understood. Currently there is no cure for IBS and no existing medication induces symptom relief in all patients. IBS-20 is a 20-herb Chinese medicinal formula that offers beneficial effects in patients with IBS; however, the underlying mechanisms are largely unknown. This study showed that IBS-20 potently inhibited LPS- or IFNΓ-stimulated expression of pro-inflammatory cytokines, as well as classically activated macrophage marker nitric oxide synthase 2. Similarly, IBS-20 or the component herb *Coptis chinensis* decreased LPS-stimulated pro-inflammatory cytokine secretion from JAWS II dendritic cells. IBS-20 or the component herbs also blocked or attenuated the IFNΓ-induced drop in transepithelial electric resistance, an index of permeability, in fully differentiated Caco-2 monolayer. Finally, the up-regulation of key inflammatory cytokines in inflamed colon from TNBS-treated mice was suppressed significantly by orally administrated IBS-20, including IFNΓ and IL-12p40. These data indicate that the anti-inflammatory activities of IBS-20 may contribute to the beneficial effects of the herbal extract in patients with IBS, providing a potential mechanism of action for IBS-20. In addition, IBS-20 may be a potential therapeutic agent against other Th1-dominant gut pathologies such as inflammatory bowel disease.

## 1. Introduction

IBS is one of the most common gastrointestinal (GI) disorders affecting up to 20% of the adult population and is characterized by chronic abdominal pain and discomfort as well as alterations in bowel habits [1]. The lack of success of the current available therapies using serotonin as a primary therapeutic target in the management of the major symptoms may be related to heterogeneity of the etiology and symptoms of IBS [2]. There is no cure for IBS and no existing medication induces symptom relief in all patients. Therefore, it is believed that new strategies of developing effective treatments for IBS should be focused on agents that can simultaneously act on multiple sites/pathways.

Chinese herbal therapies have been used to treat GI symptoms for centuries and have provided effective relief of symptoms in IBS patients. Generally, traditional herbal formulae contain many different components that act on multiple sites/pathways with potential synergistic effects and chemical reactions among the component herbs that can maximize efficacy. IBS-20 is a Chinese medicinal formula containing 20 herbs that was developed from two traditional ancient Chinese herbal formulations known as Tong Xie Yao Fang (Important Prescription for Abdominal Pain and Diarrhea) and Zhong Man Fen Xiao Wan (Separate and Reduce Fullness in the Middle). IBS-20 offered a global symptom improvement in patients with IBS in a randomized, double-blinded, placebo-controlled clinical trial [3] and is currently on a phase II dose-escalation clinical trial for IBS patient. Like many other traditional Chinese medicinal regimes, the mechanisms for the beneficial effects of IBS-20 are unknown.

There is mounting evidence indicating that previous GI infection is a key risk factor for a subgroup of IBS patients [4–7]. More recent studies also showed that some degree of immune activation is present in nearly all IBS patients tested, regardless of previous history of infection or the type of disease (constipation, diarrhea, or alternating) [4, 5, 8]. Compounds that target the immune system are an important option in the treatment of a number of GI pathologies, including inflammatory bowel disease (IBD), but have not been evaluated fully in IBS. This study was designed, therefore, to investigate, (i) the effects of IBS-20 on the cytokine production from innate immune cells stimulated with inflammatory mediators, (ii) the ability of IBS-20 to block/attenuate the dysregulated barrier function in Caco-2 cell monolayer, and (iii) the anti-inflammatory activities of IBS-20 in the murine model of TNBS-induced colitis. The results of these studies indicate that IBS-20 has potent anti-inflammatory effects on innate immune cells *in vitro* as well as on murine model of colitis *in vivo*. In addition, IBS-20 blocks/attenuates the IFN*γ*-induced disruption of epithelial barrier function. Thus, the beneficial effects of IBS-20 in patients with IBS may be attributed to the strong anti-inflammatory activities of IBS-20 or the component herbs.

## 2. Materials and Methods

### 2.1. Mice

C57BL/6 mice were purchased from The Jackson Laboratories (Bar Harbor, ME). All experiments were conducted in accordance with principles set forth in the Guide for Care and Use of Laboratory Animals, Institute of Laboratory Animal Resources, National Research Council, Health and Human Services Publication (National Institutes of Health 85-23, revised 1996), and the Beltsville Animal Care and Use Committee, 2003.

### 2.2. TNBS-Induced Colonic Inflammation in Mice

 Female, six-week-old C57BL/6 mice received intrarectal instillation of TNBS (2 mg/mouse in 40% ethanol) to induce colonic inflammation as described [9]. IBS-20 extracts were suspended in saline as the concentration of 50 mg/mL. One milliliter of solution containing 50 mg of IBS-20 extracts was orally administrated to mice (*n* = 5/group) daily started on the same day as TNBS. This dose of IBS-20 was equivalent to the human dose. Control mice were treated with 1 mL of saline and all mice were studied 7 days after treatment.

### 2.3. Plant Materials

**Figure 1:**
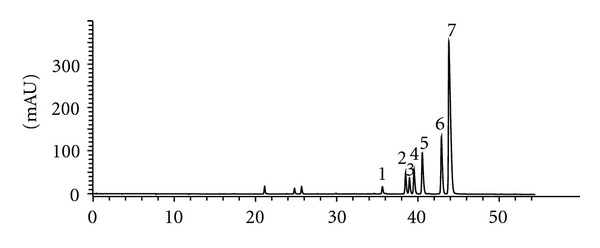
Chromatographic fingerprint of *Coptis sinensis* rhizomes. Number in the bracket is the relative retention of the peak to the marker peak: (1) 0.81, (2) 0.88, (3) 0.89, jatrorrhizine, (4) 0.90, (5) 0.93, coptisine, (6) 0.98, palmatine, (7) marker, berberine.

 The component herbs in the IBS-20 formula (Table 1) were acquired in the prescribed proportions (% w/w) through the Zhixin Chinese Pharmaceutical Co. Ltd, Guangzhou, China. Voucher samples (IBS-01 to IBS-20) have been deposited at the herbarium of the School of Chinese Medicine, The Chinese University of Hong Kong (Shatin, Hong Kong). The individual herbs were identified by their Chinese pharmaceutical as well as their scientific botanical (Latin binomial) names (Table 1) and botanically authenticated by macroscopic and microscopic means according to pharmacopoeial methods. In cases where two or more species have the same official pharmaceutical name, only one species was selected for chemical and biological/clinical studies. In addition to botanical authentication, the individual component herbs (illustrated in Figure 1 for *Coptis sinensis *rhizomes as an example) were chemically characterized by HPLC analyses employing one or more reference marker compounds to produce characteristic qualitative profiles (fingerprints). Additionally, tests for purity as measured by the contents of foreign matters, total ash, acid-insoluble ash, water, and alcohol extractives were performed according to official pharmacopoeial methods. The presence of heavy metals, pesticides, microbes, and fungal toxin (aflatoxin) contamination was also carried out and determined to meet pharmacopoeial standards as described previously [10].

**Figure 2:**
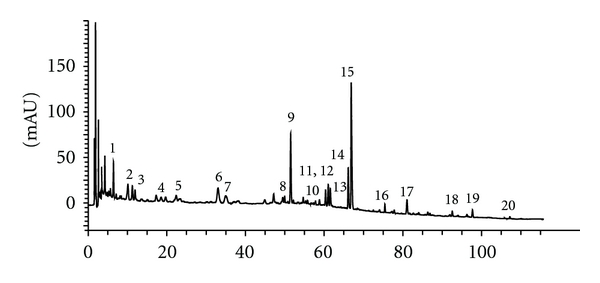
Chromatographic fingerprint of the IBS-20 formula. The identities of the peaks by LC-MS analysis are (1) Esculin, (2) Chlorogenic acid, (3) Aesculetin, (4) Paeoniflorin, (5) prim-*O*-Glucosylcimifugin, (6) Magnoflorine, (7) Liquiritin, (8) 5-*O*-Methylvisamminoside, (9) Hesperidin, (10) Columbamine, (11) Jatrorrhizine, (12) Epiberberine, (13) Coptisine, (14) Palmatine, (15) Berberine, (16) Glycyrrhizic acid, (17) Schisandrin, (18) Honokiol, (19) Magnolol, and (20) Schisandrin A.

The twenty dried component herbs were individually milled or sliced and admixed in the prescribed proportion (% w/w) as shown in Table 1, followed by extraction with water under good manufacturing practices (GMP) at the Hong Kong Institute of Biotechnology. Briefly, the herb mixture, totaling 400 kg, was decocted with 10-fold (w/v) of boiling distilled water for 60 minutes, cooled and collected. Fresh boiling water was added to the marc and decocted for a second time. The cooled extracts were pooled, filtered, concentrated, and spray dried to obtain a powder (34% yield w/w based on raw herbs). An aliquot was set aside for chemical and preclinical biological studies. The remaining portion was formulated with water-soluble starch (excipient) into the clinical product. The HPLC fingerprint of IBS-20 (Figure 2) and the content of selected marker compounds were determined as previously described [10]. For *in vitro* studies, the herbal extracts were processed for removal of possible contaminating LPS using Detoxi-Gel Endotoxin Removing Gel (Thermo Scientific, Rockford, IL).

### 2.4. Reference Marker Compounds and Reagents

Reference marker compounds (Table 1) for qualitative and quantitative HPLC analyses were obtained from the National Institute for the Control of Pharmaceutical and Biological Products (Beijing, China) and were subjected to identity validation by MS and NMR analyses and purity (>98%) analysis by HPLC-DAD and/or LC-MS.

### 2.5. Preparation of Bone Marrow-Derived Macrophages (BMDM) and Cell Culture

 Nonadherent bone marrow mononuclear cells were obtained from mice by flushing the marrow from tibia and femurs into HyClone MEM Alpha medium (Thermo) containing 10% FBS. Cells were cultured overnight at 37°C with 5% CO_2_ in HyClone MEM Alpha medium to deplete adherent stromal cells. The nonadherent mononuclear cells were collected, treated with Red Blood Cell Lysis Buffer to deplete red blood cells. These cells were then cultured in the presence of 20 ng/mL recombinant mouse M-CSF (R&D Systems, Minneapolis, MN) for 7 days to generate fully differentiated macrophages with the medium changed every other day. JAWS II (CRL-11904, ATCC), a murine dendritic cell line, was cultured in the Alpha minimum essential medium with 20% FBS, 1% P/S antibiotic, 4 mM glutamine, 1 mM sodium pyruvate, and 5 ng/mL murine GM-CSF. Macrophages and dendritic cells (DCs) were plated in 6-well plates and treated in triplicates with the herbal extracts of IBS-20 or the individual components herbs in the presence or absence of inflammatory mediators LPS, IFN*γ*, or both. After 24 hours, cells were collected in Trizol for RNA isolation while cell-free supernatants were collected Luminex Assay on cytokine release. Based on our preliminary experiments, we selected the most appropriate concentration of LPS for the individual experiments as follows: 1 ng/mL for cytokine mRNA expression of BMDM, 10 ng/mL for cytokine release of BMDM, and 2 ng/mL for JAWS II cells.

### 2.6. Microsnap Well Assay for Transepithelial Electrical Resistance (TEER)

 Caco-2 cells (HTB-37, ATCC), a human epithelial cell line, were cultured in Eagle's minimum essential medium with 20% FBS. 5 × 10^5^ cells per well are seeded on a 12-well transwell plate with 12 mm insert (Costar 3460, Corning Incorporated) and incubated at 37°C with 5% CO_2_. The bottom of the well (basolateral side) was filled with 1.5 mL culture medium and top chamber (apical side) with 0.5 mL medium. Cells were a confluent polarized epithelial monolayer after 10–14 days in culture. The monolayer was then treated with herbal extracts of IBS-20 or the individual component herbs added to both apical and basolateral sides in the presence or absence of IFN-*γ* (added to the basolateral side), a well-recognized epithelial barrier disruptor. TEER was monitored daily before the treatment and then again at 24 and 48 hours after treatment.

### 2.7. RNA Extraction, cDNA Synthesis, and Real-Time Quantitative PCR

Total RNA was extracted from cultured cells or colon whole tissue as described previously [11]. RNA samples (2 mg) were reverse-transcribed to cDNA using the First Strand cDNA Synthase Kit (MBI Fermentas, Hanover, MD) with random hexamer primer. Real-time quantitative PCR (qPCR) was performed on an iCycler detection system (Bio-Rad, Hercules, CA) as described. The fold changes in mRNA expressions for targeted genes were relative to the respective vehicle groups of mice after normalization to 18S rRNA. Primers for qPCR were designed by Beacon Designer 7.0 (Premier Biosoft International, Palo Alto, CA) and synthesized by Sigma or the Biopolymer Laboratory of the University of Maryland (Baltimore, MD). The sequences were listed in Table 2 or as described previously [11].

### 2.8. Luminex Assay for Cytokine Release

 Cell-free supernatant was collected and the cytokine release was determined using Luminex 100 System in the Cytokine Core Lab of the University of Maryland (Baltimore, MD).

### 2.9. Data Analysis

Statistical analysis was performed using one-way ANOVA followed by Newman-Keuls test to compare the responses and gene expression. Appropriate vehicle-, time-, and age-matched controls were also included throughout the studies.

## 3. Results

### 3.1. IBS-20 Potently Inhibits the Expression/Production of Pro-Inflammatory Cytokines in Macrophages

**Figure 3:**
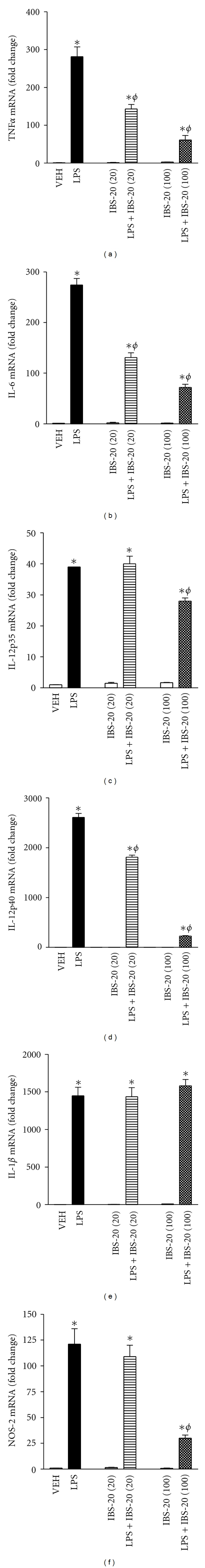
IBS-20 inhibited LPS-stimulated expression of pro-inflammatory cytokines/mediators in bone marrow-derived macrophages (BMDMs). BMDMs were treated with LPS (1 ng/mL) in the presence or absence of IBS-20 extracts (20 or 100 *μ*g/mL). After 24 hours, cells were collected in Trizol for mRNA expression of TNF*α* (a), IL-6 (b), IL-12p35 (c), IL-12p40 (d), IL-1*β* (e), and NOS-2 (f) by qPCR. *P < 0.05 versus respective vehicle (VEH), ^*ϕ*^ *P* < 0.05 versus LPS alone. Data shown in bar graphs are the mean ± s.e.m and representative of at least two independent experiments.

**Figure 4:**
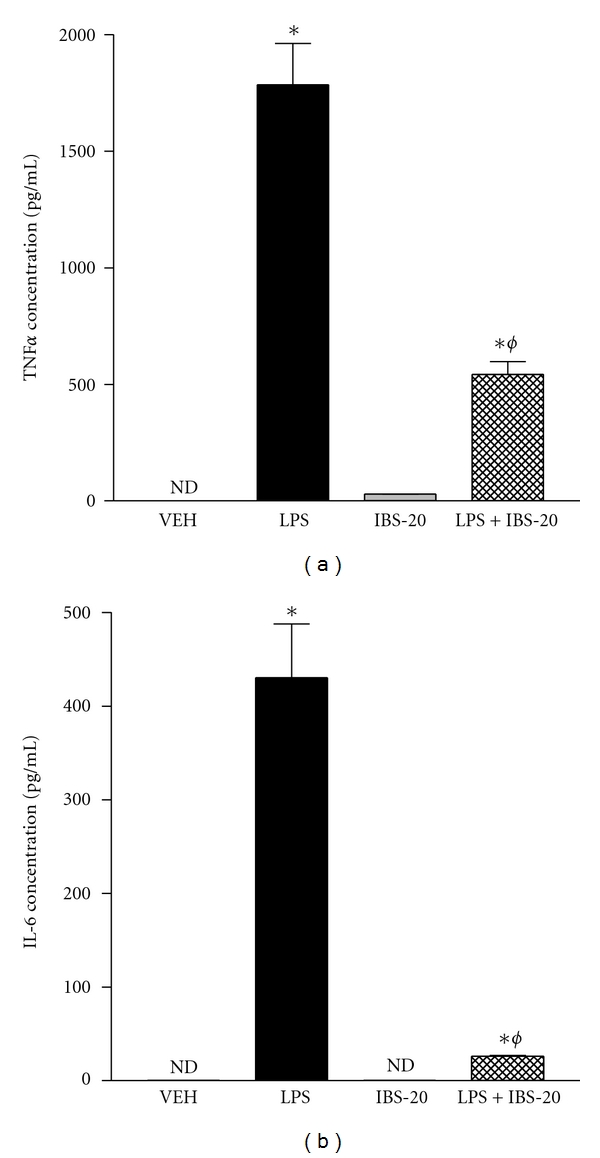
IBS-20 inhibited cytokines release of TNF*α* and IL-6 from BMDMs. BMDMs were treated with LPS (10 ng/mL) in the presence or absence of IBS-20 extracts (0.1 mg/mL). After 24 hours, cell-free supernatants were collected for cytokine release of TNF*α* (a) and IL-6 (b) by Luminex Assay. ND: not detected. *P < 0.05 versus vehicle (VEH) or IBS-20; ^*ϕ*^ *P* < 0.05 versus LPS. Data shown in bar graphs are the mean ± s.e.m and representative of at least two independent experiments.

Given the fact that low-grade inflammation is often present in patients with IBS and many Chinese herbs possess anti-inflammatory properties, we sought to determine if IBS-20 can inhibit the pro-inflammatory cytokine production from macrophages, one of the major cells involved in inflammation. We performed studies in bone marrow-derived macrophages (BMDMs) that are generally considered to be a pure population of mature macrophages and often serve as a valuable cell model for assessing anti-inflammatory activities [12]. Three different concentrations of IBS-20 were initially tested: 20, 100, and 300 *μ*g/mL. IBS-20 had no effect on basal cytokine expression or cell viability at the concentrations of 20 and 100 *μ*g/mL (Figure 3) but caused some degrees of cell death at 300 *μ*g/mL. IBS-20 at 20 *μ*g/mL had modest inhibitory effects on LPS-stimulated up-regulation of TNF*α*, IL-6, and IL-12p40, a subunit shared by both IL-12 and IL-23 (Figures 3(a), 3(b), and 3(d)). At 100 *μ*g/mL, IBS-20 potently inhibited LPS-stimulated up-regulation of several major pro-inflammatory cytokines, including IL-6, TNF*α*, IL-12p35, and IL-12p40 (Figures 3(a)–3(d)). In contrast, IBS-20 did not affect the basal or LPS-stimulated cytokine expression of IL-1*β* (Figure 3(e)), IFN*γ*, IL-23p19, or IL-13 (data not shown). It also was without effect on the anti-inflammatory cytokines, TGF*β* or IL-10 (data not shown). These date indicate that IBS-20 is not a general anti-inflammatory agent, but rather an inhibitor of specific cytokines/mediators. Changes in mRNA expression do not always translate into changes in protein expression; therefore, we measured cytokine release using Luminex Assay. Consistently, the LPS-stimulated cytokine release of TNF*α* and IL-6 from BMDM was significantly less when treated with IBS-20 (Figure 4), indicating that IBS-20 inhibits the pro-inflammatory cytokines at both translational and transcriptional levels. Notably, IL-12p40 protein was not detectable in the cell culture supernatant of BMDM even after stimulated with LPS (1 ng/mL) for 24 hours (data not shown).

It is well established that macrophages undergo distinct pathways of activation under different cytokine microenvironments [13]. In the presence of LPS, macrophages undergo classical activation (CAM*ϕ*) characterized by the significant up-regulation of inducible nitric oxide synthase (NOS-2) expression, the specific molecular marker for inflammatory CAM*ϕ*. IBS-20 significantly attenuated the LPS-induced up-regulation of NOS-2 in BMDM (Figure 3(f)), indicating that IBS-20 can also influence the phenotypic development of macrophages.

**Figure 5:**
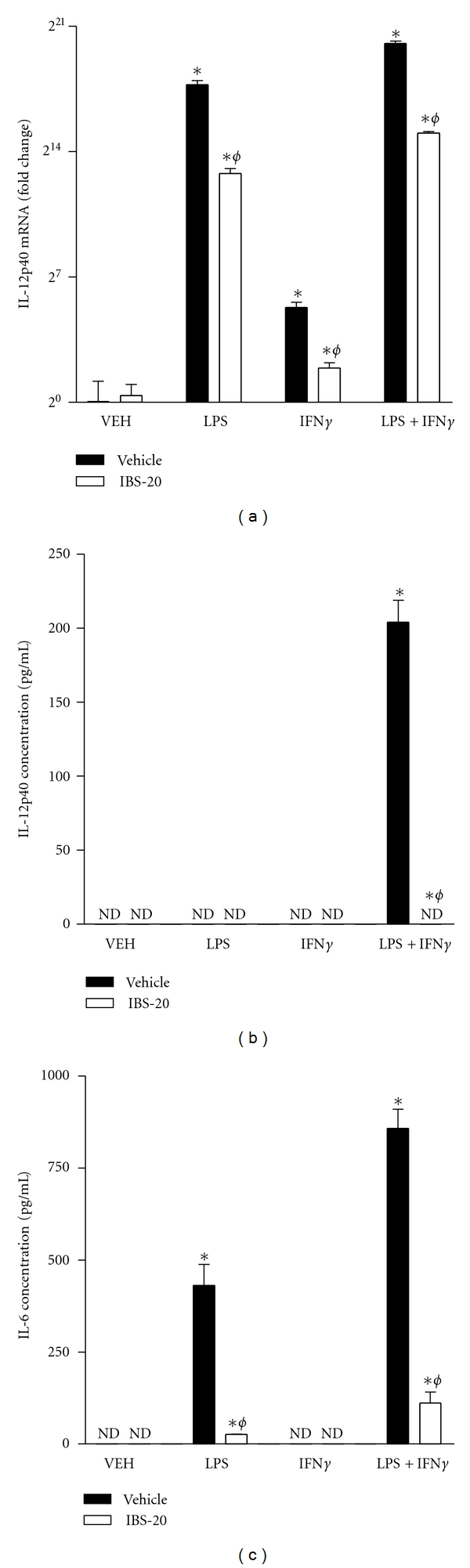
IBS-20 inhibited the cytokine expression/release from BMDMs stimulated with inflammatory mediators. BMDMs were treated with LPS (10 ng/mL), IFN*γ* (10 ng/mL), or both in the presence or absence of IBS-20 extracts (0.1 mg/mL). After 24 hours, cells were collected in Trizol for mRNA expression of IL-12p40 by qPCR (a). Cell-free supernatants were collected for cytokine release of IL-12p40 (b) or IL-6 (c) by Luminex Assay. ND: not detected. *P < 0.05 versus respective VEH; ^*ϕ*^ *P* < 0.05 versus respective vehicle. Data shown in bar graphs are the mean ± s.e.m and representative of at least two independent experiments.

IFN*γ* is an important pro-inflammatory cytokine produced mainly by Th1 and antigen-presenting cells (macrophage and DC). Increased levels of IFN*γ* are implicated in various inflammatory pathologies. To investigate whether IBS-20 can affect the biological activities of IFN*γ*, we stimulated BMDM with IFN*γ*, LPS, or IFN*γ* + LPS in the presence or absence of IBS-20. qPCR showed that IFN*γ* or LPS alone upregulated the mRNA expression of IL-12p40 and, when given together, had a synergetic effect (Figure 5(a)). The presence of IBS-20 significantly inhibited the IL-12p40 expression in BMDM stimulated with LPS or IFN*γ* alone, or in combination. Confirming the qPCR results, Luminex assay of cell culture supernatant showed that IBS-20 completely blocked LPS + IFN*γ*-induced IL-12p40 release (Figure 5(b)). Similarly, IBS-20 greatly decreased the IL-6 production in macrophages stimulated with both LPS and IFN*γ* (Figure 5(c)).

 The biological activities of LPS depend on TLR4, a toll-like receptor implicated in Th1-dominant inflammation. To further explore the mechanisms by which IBS-20 inhibits LPS-stimulated cytokine production, we measured the expression of TLR4. qPCR showed that TLR4 expression was unaffected by either LPS or IBS-20 treatment (data not shown), indicating that the anti-inflammatory activities of IBS-20 do not involve TLR4.

### 3.2. IBS-20 Potently Inhibits the Expression/Production of Proinflammatory Cytokines in JASW-II Cells

**Figure 6:**
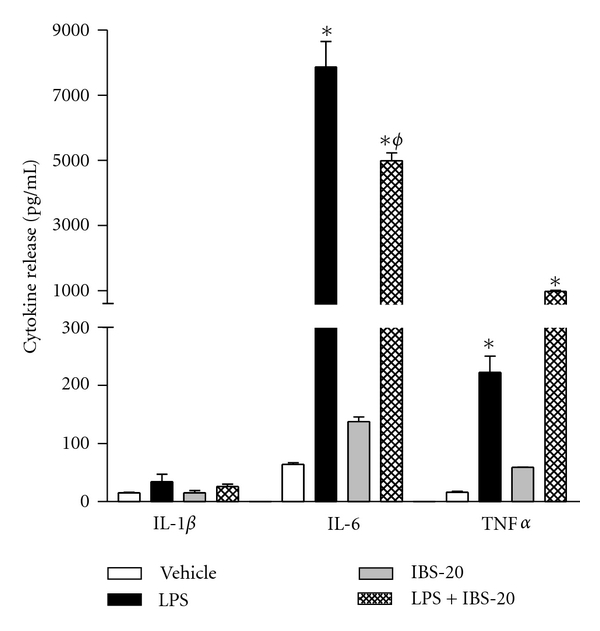
Effects of IBS-20 on cytokine secretion of JAWS II cells. Cells were treated with IBS-20 extracts (0.1 mg/mL) in the presence or absence of LPS (2 ng/mL) and the cell-free supernatants were collected after 24-hour treatment. Cytokine secretion of IL-1*β*, IL-6, and TNF*α* was determined by Luminex Assay. *P < 0.05 versus respective vehicle or IBS-20; ^*ϕ*^ *P* < 0.05 versus IBS-20. Data shown in bar graphs are the mean ± s.e.m and representative of at least two independent experiments.

 Dendritic cells represent another type of major innate immune cells important for adaptive immunity and aberrant activation of DC implicated in variety of inflammation. To determine whether IBS-20 affects the activities of DC, we performed studies on JASW-II cells, a mouse dendritic cell line. Cells were treated with IBS-20 in the presence or absence of LPS and cell culture supernatants were collected for Luminex assay of pro-inflammatory cytokine release. Consistent with its anti-inflammatory activities on macrophages, IBS-20 significantly decreased LPS-induced IL-6 secretion from JAWS-II cell (Figure 6). Again, the effect on IL-1*β* production was not apparent due to the very low expression level even after stimulation with LPS (Figure 6). On the other hand, LPS-stimulated TNF*α* secretion was not suppressed by IBS-20 (Figure 6).

### 3.3. Coptis Chinensis Is an Anti-Inflammatory Component Herb of IBS-20

**Figure 7:**
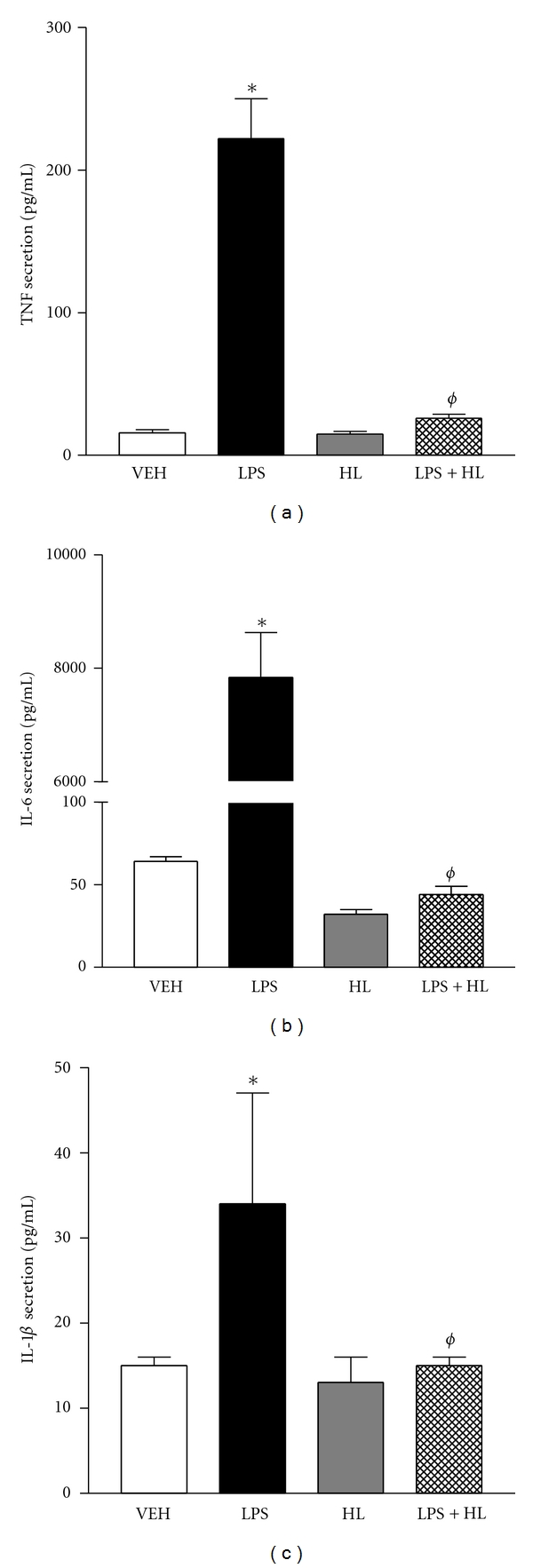
Inhibitory effects of *Coptis chinensis* (Huang Lian, HL) on the cytokine secretion of JAWS II cells. Cells were treated with *Coptis chinensis* extracts (20 *μ*g/mL) in the presence or absence of LPS (2 ng/mL) and the cell-free supernatants were collected after 24-hour treatment. Cytokine secretion of TNF*α* (a), IL-6 (b), and IL-1*β* (c) was determined by Luminex Assay. *P < 0.05 versus respective VEH; ^*ϕ*^ *P* < 0.05 versus HL.

IBS-20 is an herbal formula consisting of 20 component herbs. Among the several herbs that have documented anti-inflammatory activities [14], we selected *Coptis chinensis* (Huang Lian, HL) for further testing the effects on LPS-stimulated cytokine release from JAWS II cells. Although it had no effect on the basal level of cytokine release, *Coptis chinensis* almost completely abolished the LPS-stimulated cytokine release of pro-inflammatory IL-6, IL-1*β*, and TNF*α* (Figures 7(a)–7(c)).

### 3.4. IBS-20 or the Selected Component Herbs Attenuate/Block the IFN*γ*-Induced Epithelial Barrier Dysfunction

**Figure 8:**
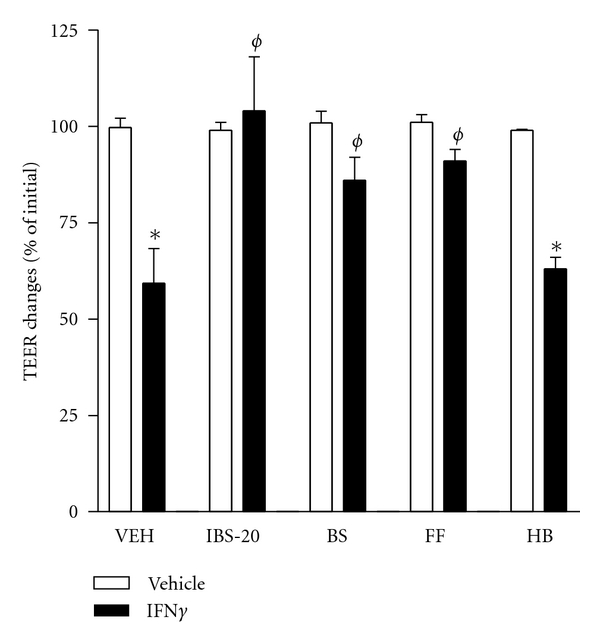
Effects of IBS-20 or the component herbs on barrier function of fully differentiated Caco-2 monolayer. 5 × 10^5^ cells per well were seeded on a 12-well transwell plate with 12 mm insert and cultured for 10–14 days. The polarized epithelial cell monolayer was then treated with herbal extracts of IBS-20 (100 *μ*g/mL) or the individual component herbs (20 *μ*g/mL), *Paeonia lactiflora* Pall (Bai Shao, BS), *Saposhnikovia divaricata* Schischk (Fang Feng, FF), and *Phellodendron amurense* (Huang Bai, HB), added to both apical and basolateral sides in the presence or absence of IFN*γ* (20 ng/mL, added to the basolateral side). TEER was measured at 48 hours after treatment. *P < 0.05 versus respective; ^*ϕ*^ *P* < 0.05 versus VEH-IFN*γ*. Data shown in bar graphs are the mean ± s.e.m and representative of at least two independent experiments.

Epithelial cells in the mucosal surface form a monolayer barrier that limits the exposure of pathogens or products to the underlying immune cells; therefore, maintaining an intact mucosal barrier is arguably one of the most critical factors in immune homeostasis. Having established the anti-inflammatory activities of IBS-20 in the innate immune cells, we next determined if IBS-20 was capable of influencing epithelial barrier function. We cultured Caco-2 cells, a human colonic epithelial cell line, and treated them with IBS-20 in the presence of IFN*γ*, a well-established epithelial barrier disruptor. Transepithelial electric resistance (TEER) was measured as an index of permeability. Consistent with previous reports, IFN*γ* effectively decreased TEER of Caco-2 monolayer after 48-hour treatment indicating an increase in permeability (Figure 8). The presence of IBS-20 prevented this IFN*γ*-induced decrease in TEER, indicating maintenance of normal barrier function (Figure 8). Two of the individual herbs, *Paeonia lactiflora* Pall (Bai Shao, BS) and *Saposhnikovia divaricata* Schischk (Fang Feng, FF), also significantly attenuated the IFN*γ*-induced drop in TEER, while *Phellodendron amurense* (Huang Bai, HB) had no effect (Figure 8). It is noteworthy that neither IBS-20 nor any of the individual component herbs tested had any effect on the basal TEER.

### 3.5. IBS-20 Attenuates the TNBS-Induced Upregulation of Proinflammatory Cytokines in Colon

**Figure 9:**
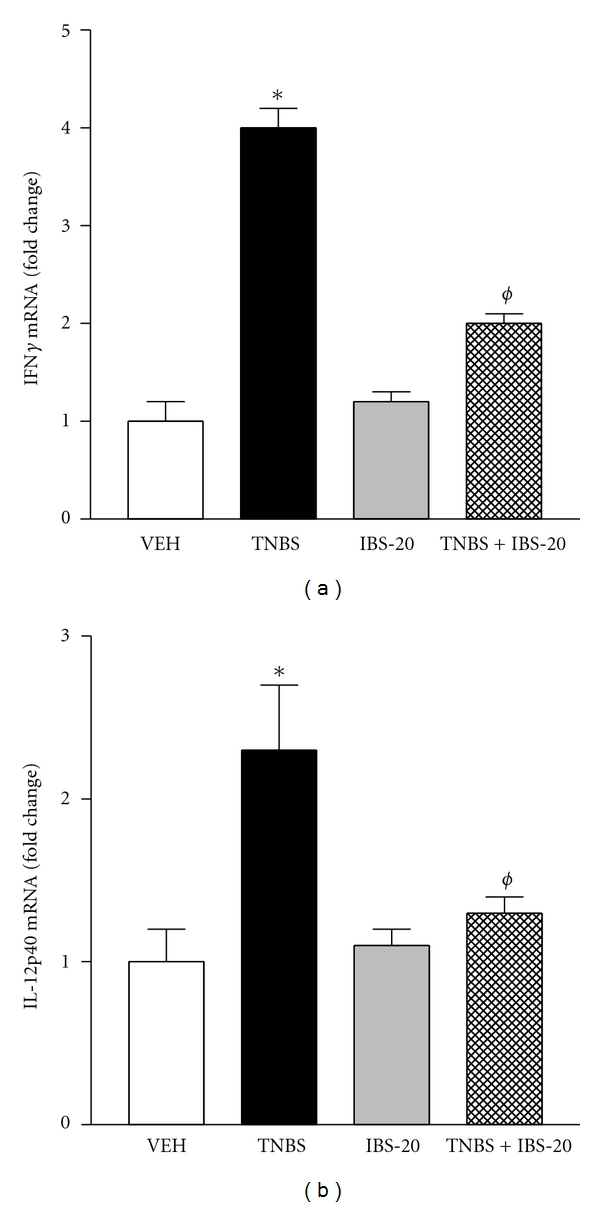
Administration of IBS-20 suppresses the up-regulation of pro-inflammatory cytokine expression in the inflamed colon *in vivo*. Mice received intrarectal instillation of TNBS (2 mg/mouse in 40% ethanol) to induce colonic inflammation. IBS-20 extracts were given to mice daily via gavage. Mice were euthanized at day 7 after TNBS, and the colon was collected for mRNA expression of cytokines by qPCR. Results on the expression of IFN*γ* (a) and IL-12p40 (b) were shown. *P < 0.05 versus VEH; ^*ϕ*^ *P* < 0.05 versus IBS-20; *n* = 5 for each group.

The anti-inflammatory activities of IBS-20 *in vitro* in both immune and epithelia cells suggest that IBS-20 may provide beneficial effect for inflammatory disease. We extended our investigation to the murine model of TNBS-induced colonic inflammation. Beginning the day of intracolonic administration of TNBS, mice were given daily IBS-20 for 7 days via gavage. The TNBS-induced macroscopic (tissue edema, length, and weight of colon) and histological abnormalities (crypt damage and inflammatory cell infiltration) of colon were unchanged by IBS-20 (data not shown); however, up-regulation of two key Th1 cytokines, IFN*γ* and IL-12p40, in inflamed colon was attenuated significantly in mice receiving IBS-20 (Figure 9).

## 4. Discussion

Herbal therapy has been used in Chinese Medicine for many thousands of years and has regained popularity over the last few decades worldwide. Their efficacy and mechanisms of actions, however, remain largely unknown. The current study shows that IBS-20, a multicomponent Chinese medicinal formula developed from traditional ancient Chinese herbal formulations, has potent anti-inflammatory activities in both innate immune cells *in vitro* as well as the TNBS-inflamed colon *in vivo*. In addition, the herbal formula can protect the colonic epithelial monolayer from the inflammatory mediator-induced barrier dysfunction.

The gut harbors the largest immune system in the body and the mucosa is considered to be the initial site of interaction with commensal and pathogenic organisms [15]. Even though gut homeostasis is under tight control, immune dysregulation occurs and contributes to a variety of inflammatory pathologies [16]. IBS is the most commonly diagnosed gastrointestinal disorder, and recent work implicates a role of inflammation in the pathogenesis of IBS [4, 5, 8]. Indeed, increased levels of pro-inflammatory and/or decreased levels of anti-inflammatory cytokines are found in the peripheral blood mononuclear cells, circulation, and/or mucosal samples in patients with IBS [4, 5, 17]. In addition, increased infiltration of immune cells (mostly mast cell and T lymphocytes) [18–20] or abnormal activation of T/B cell [8, 21] are observed in the mucosa of patients with IBS. Through the release of pro-inflammatory mediators, these immune cells interact with, and sensitize, the sensory and motor neurons, leading to colorectal hypersensitivity detected in many IBS patients [22, 23].

As one of the major players in gut immunity, macrophages are present constitutively in the intestinal lamina propria [24–26]. Under normal conditions, intestinal macrophages lack or have reduced responses to inflammatory stimuli. During active disease, the number of macrophages is increased in the lamina propria as a result of recruitment of circulating monocytes. These recruited macrophages release large amounts of pro-inflammatory cytokines, such as IL-1*β*, IL-12, TNF*α*, and IFN*γ*, which are important in both the onset and development of inflammation [25]. Finally, macrophages can influence the differentiation and expansion of inflammatory Th1/Th17 effector cells by releasing IL-12 and IFN*γ* [26, 27]. In this study, IBS-20 potently inhibited production of pro-inflammatory cytokines from BMDM stimulated by inflammatory mediators such as LPS and IFN*γ*.

Macrophages express many types of TLRs, including TLR4 which is responsible for LPS stimulation of large amounts of pro-inflammatory cytokine production [28, 29]. Increased expression of TLR is reported in the colonic mucosa of animal models of IBS [27]. IBS-20 did not affect the TLR-4 expression in macrophages, indicating that the intracellular signaling molecules of TLR-4, rather than the receptor itself, are the potential target of IBS-20. LPS activates TLR4 signaling by binding to TLR4 : MD2 receptor complex, a process facilitated by the coordination of LPS binding protein and CD14. It then leads to the activation of MyD88-dependent and/or independent intracellular signaling pathway. The MyD88-dependent pathway causes rapid activation of transcription factors NF-*κ*B and AP-1, and is responsible for the early production of pro-inflammatory cytokines such as IL-1*β* and TNF*α*. The MyD88-independent pathway, on the other hand, activates IRF3 and is responsible for the induction of Type 1 interferons and interferon-inducible genes that are important for antiviral and antibacterial responses. The distinct effect of IBS-20 on LPS-stimulated cytokine expression suggests that the herbs may influence multiple sites/pathways of TLR-4 intracellular signaling to inhibit the LPS-induced pro-inflammatory cytokine production, although more details remain to be fully understood.

Dendritic cells are another major type of innate immune cells and professional antigen presenting cells that play a crucial role in both innate and adaptive immunity. Intestinal DCs reside in mucosa or lymphoid tissues such as Peyer's patches and mesenteric lymph nodes. Through sampling and processing numerous luminal antigens from food, microorganisms, and their products, mucosal DCs are important for the immune system to achieve tolerance and maintain homeostasis. Abnormal DC functions have been associated with intestinal inflammation. The current study showed that in addition to its anti-inflammatory effects on macrophages, IBS-20 strongly inhibited LPS-stimulated cytokine production from JASW II cells, a DC cell line, suggesting a global anti-inflammatory activity of IBS-20 that is likely mediated by antigen presenting cells.* Coptis chinensis *is among the several component herbs of IBS-20 that have well documented anti-inflammatory activities. Previous studies show that *Coptis chinensis* extracts or the major alkaloid from* Coptis chinensis* can inhibit the expression of various pro-inflammatory cytokines such as IL-6 and TNF*α* [14, 30, 31]. Consistently, our study showed that *Coptis chinensis *aqueous extracts completely blocked LPS-stimulated cytokine release of IL-6, IL-1*β*, and TNF*α* from DC, indicating that *Coptis chinensis *is the component herb that contributes, at least in part, to the anti-inflammatory action of IBS-20. It remains to be determined whether any of other individual herbs has similar effects.

Control of intestinal permeability is critical to host defense because enhanced permeability facilitates passage of large numbers of intraluminal bacteria, antigens, or other pathogen-generated molecules across the mucosal barrier that trigger immune activation [32]. Evidence indicates that IBS patients exhibit increased mucosal permeability in the small intestine or colon irrespective of IBS subtype, and that is often correlated with the disease severity [32–35]. In addition, changes in mucosal barrier function and the resulting immune activation are involved in triggering visceral hypersensitivity in patients with IBS [18, 22, 33]. This study showed that IBS-20 or the component herbs are able to preserve normal mucosal barrier function, providing another mechanism for the beneficial effects in patients. It would be interesting to identify the molecular targets of IBS-20 acting through the intracellular signaling and tight junction proteins that may contribute to herbal effects on epithelial barrier function.

TNBS-induced colonic inflammation in mice is a commonly used chemically induced model that features up-regulation of pro-inflammatory cytokines (IL-1*β*, IFN*γ*, TNF*α*, IL-12, IL-17A). This model has been used for investigating the pathogenesis of IBD and testing the anti-inflammatory activities of agents with therapeutic potential [36]. Macrophage activation with increased levels of Th1 cytokines in the colonic mucosa is another feature of mice treated with TNBS. In addition, TNBS treatment mimics several key aspects of symptoms in patients with IBS, including the increase in mucosal permeability and visceral hypersensitivity in response to colorectal distension [37]. The underlying mechanisms for the hyperalgesia are not fully understood but may involve the release of pro-inflammatory mediators interacting with, and sensitizing, the sensory and motor neurons [38]. Administration of IBS-20 with TNBS simultaneously abolished or significantly attenuated the up-regulation of pro-inflammatory cytokines in the inflamed colon consistent with its anti-inflammatory activities. It was surprising to see that the clinical symptoms or histological abnormalities of the colon in TNBS-treated mice were not improved by IBS-20. This could be attributed to a number of factors relating to the use of IBS-20 *in vivo* including the inability to inhibit the TNBS-induced epithelial barrier breakdown or to block production of IL-1*β*, a major pro-inflammatory cytokine implicated in TNBS-induced colonic inflammation.

Collectively, this study demonstrates that IBS-20 possesses strong anti-inflammatory properties. The herbal extracts not only inhibit the pro-inflammatory cytokine production from the immune cells but also block the inflammatory mediator-induced epithelial cell barrier disruption. Moreover, IBS-20 is capable of suppressing the up-regulation of inflammatory cytokine in the inflamed colon *in vivo*. Thus, the beneficial effects of IBS-20 in patients with IBS may be attributed to its anti-inflammatory activities. It should be noted that IBS patients are heterogeneous and inflammation may only occur in certain subtypes of patients particularly those with histories of previous infection. These factors should be taken into account when applying anti-inflammatory therapies to IBS patients. Given the fact that there are similarities and clinical overlaps between IBS and other gut inflammatory diseases [39], IBS-20 may also be a potential therapeutic agent against other Th1-dominant gut pathologies such as IBD.

## Figures and Tables

**Table 1:** Component herbs in the IBS-20 formula and their reference markers.

Pharmaceutical name and Chinese phonetic name	Botanical name and plant part	Composition (%)	Reference marker(s)	
			Qualitative analysis (HPLC fingerprinting)	Quantitative analysis (HPLC)
Angelicae Dahuricae Radix (Bai Zhi)	* Angelica dahurica* (Fisch.ex Hoff.) Benth. et Hook. f. (Apiaceae); Root	2	Imperatorin	Imperatorin
Artemisiae Scopariae Herba (Yin Chen)	*Artemisia scoparia* Waldst. et Kit. (Asteraceae); Aerial part	13	Chlorogenic acid	Chlorogenic acid
Atractylodis Macrocephalae Rhizoma (Bai Zhu)	*Atractylodes macrocephala* Koldz. (Asteraceae); Rhizome	9	—	—
Aucklandiae Radix (Mu Xiang)	*Aucklandia lappa* Decne. (Asteraceae); Root	3	Costunolide	Costunolide; Dehydrocostus lactone
Bupleuri Radix (Chai Hu)	*Bupleurum chinense* DC. (Apiaceae); Root	4.5	Saikosaponin a, d	Saikosaponin a
Citri Reticulatae Pericarpium (Chen Pi)	*Citrus reticulata* Blanco (Rutaceae); Ripe fruit pericarp	3	Hesperidin	Hesperidin
Codonopsis Radix (Dang Shen)	*Codonopsis pilosula* Nannf. Var. modest a (Nannf.) L. T. Shen (Campanulaceae); Root	7	Lobetyolin	Lobetyolin
Coicis Semen (Yi Yi Ren)	*Coix lacryma-jobi* L.var.*ma-yuen *(Roman.) Stapf. (Poaceae); Ripe kernel	7	Glycerol trioleate	Glycerol trioleate
Coptidis Rhizoma (Huang Lian)	*Coptis chinensis* Franch. (Ranunculaceae); Rhizome	3	Berberine	Berberine; Palmatine
Fraxini Cortex (Qin Pi)	*Fraxinus rhynchophylla* Hance (Oleaceae); Branch or stem bark	4.5	Aesculetin	Aesculetin; Esculin
Glycyrrhizae Radix et Rhizoma Praeparata cum Melle (Zhi Gan Cao)	*Glycyrrhiz uralensis *Fisch.(Fabaceae); Root and rhizome	4.5	Glycyrrhizic acid	Glycyrrhizic acid
Magnoliae Officinalis Cortex (Hou Po)	*Magnolia officinalis* Rehd. Et Wils. (Magnoliaceae); Root, branch and stem bark	4.5	Magnolol	Magnolol; Honokiol
Paeoniae Alba Radix (Bai Shao)	*Paeonia lactiflora* Pall. (Paeoniaceae); Root	3	Paeoniflorin	Paeoniflorin
Plantaginis Semen (Che Qian Zi)	*Plantago asiatica* L. (Plantaginaceae); Ripe seed	4.5	—	—
Phellodendri Amurensis Cortex (Guan Huang Bo)	*Phellodendron amurense* Rupr. (Rutaceae); Bark	4.5	Berberine	Berberine; Palmatine
Pogostemonis Herba (Guang Huo Xiang)	*Pogostemon cablin* (Blanco) Benth.(Lamiaceae); Aerial part	4.5	—	Patchouli alcohol (GC method was used for analysis)
Poria (Fu Ling)	*Poria cocos* (Schw.) Wolf (Polyporaceae); Sclerotium	4.5	—	—
Saposhnikoviae Radix (Fang Feng)	*Saposhnikovia divaricata* (Turcz.) Schischk. (Apiaceae); Root	3	5-*O*-Methylvisammioside	prim-*O*-Glucosylcimifugin; 5-*O*-Methylvisammioside
Schisandrae Chinensis Fructus (Wu Wei Zi)	*Schisandra chinensis* (Turcz.) Baill. (Schisandraceae); Ripe fruit	7	Schisandrin	Schisandrin
Rhizoma Zingiberis Praeparatum (Pao Jiang)	*Zingiber officinale* Rose (Zingiberaceae); Prepared rhizome	4.5	6-Gingerol	6-Gingerol

**Table 2:** Primer sequences for real-time quantitative PCR.

Gene	Primer sequences (5′ to 3′)
IFN*γ*	Forward, TGGCTGTTTCTGGCTGTTACTG
	Reverse, AGGTGTGATTCAATGACGCTTATG
IL-1*β*	Forward, TCTATACCTGTCCTGTGTAATG
	Reverse, GCTTGTGCTCTGCTTGTG
IL-6	Forward, TCCATCCAGTTGCCTTCTTG
	Reverse, TTTCTCATTTCCACGATTTCCC
IL-10	Forward, TCTCCCCTGTGAAAATAAGAG
	Reverse, GCCTTGTAGACACCTTGG
IL12p35	Forward, TTGATGATGACCCTGTGCCTTGG
	Reverse, GATTCTGAAGTGCTGCGTTGATGG
IL-12p40	Forward, TGAGAACTACAGCACCAGCTTCTT
	Reverse, CTTCAAAGGCTTCATCTGCAAGT
IL-23p19	Forward, CAACTTCACACCTCCCTAC
	Reverse, CCACTGCTGACTAGAACTC
NOS-2	Forward, CGGAGCCTTTAGACCTCAACA
	Reverse, CCCTCGAAGGTGAGCTGAAC
TGF-*β*	Forward, CTAATGGTGGACCGCAACAAC
	Reverse, GCACGGGACAGCAATGGG
TLR4	Forward, ACCAAGAACATAGATCTGAGCTTCAA
	Reverse, ATGCCATGCCTTGTCTTCAATT
TNF*α*	CCTCCCTCTCATCAGTTCTATGG
	Reverse, GGCTACAGGCTTGTCACTCG
